# Analyzing international airtime top-up transfers for migration and mobility

**DOI:** 10.1007/s41060-023-00396-7

**Published:** 2023-05-25

**Authors:** Bilgeçağ Aydoğdu, Hanif Samad, Shiqi Bai, Sami Abboud, Ilias Gorantis, Albert Ali Salah

**Affiliations:** 1 Information and Computing Sciences, Utrecht University, Princetonplein 5, 3584 CC Utrecht, The Netherlands; 2 DT One, 1 Raffles Place, Tower 2, Singapore, 048616 Singapore; 3 Department of Computer Engineering, Boğaziçi University, 34342 Bebek, Istanbul, Turkey

**Keywords:** Airtime top-up transfers, Migration, Mobile phone data, Remittances

## Abstract

International airtime top-up transfers enable prepaid mobile phone users to send top-ups and data bundles to users in other countries, as well as make payments, in real time. These are heavily used by migrants to financially assist their families in their home countries and consequently could be a valuable source of information for migration and mobility analysis. However, top-up transfers are understudied as a form of money remittance in migration. In this paper, we explore the determinants and the potential of top-up transactions to complement remittance and migration statistics. Our results show that such data can provide insights into migrant groups, particularly for irregular migration and for estimating the real-time distribution of migrant groups for a given country.

## Introduction

Airtime top-up transfers are mobile phone credit transfers between two phone users. These are usually sent in small amounts to purchase data, SMS, or call minutes, as well as to pay phone bills and to purchase other services. In the last decades, the airtime top-ups have been gaining prominence, thanks to increasing mobile phone ownership in developing countries [37]. International top-up transfers have become increasingly prevalent in the airtime market, with increasing numbers of intermediary service providers and higher user demand.

International Monetary Fund (IMF) classifies airtime top-up transfers as one of the transaction channels for “remittances” [23], which is money sent home by migrants, and as such, a very important source of financial inflow to developing countries. Since in many developing countries, top-up transfers tend to be less expensive than other money sending options (such as banks, or post offices) [45], these constitute a convenient remittance channel for migrants supporting their relatives in their home countries [11]. Top-up transfers can be used for services and purchases in some countries [31, 49]. A large percentage of mobile phones in developing countries are prepaid [38], which contributes to the popularity of top-up transfers.

In this paper, we explore the determinants and the potential of top-up transactions to complement remittance and migration statistics. This is an understudied data source in migration research, as such data are difficult to obtain. We work with one of the major companies that provides top-up transfer services between telecommunications operators in different countries, which operates in over 160 countries. Each pair of sending and receiving countries is called a “corridor”, and while we do not have data on all possible corridors (depending on the availability of the services in particular countries), valuable insights can be obtained for the existing corridors, such as the distribution of the migrants from a receiving country within the sending country.

We ask two main questions in this paper: (1) What are the factors influencing the volume of top-up transfers for a specific corridor? (2) What can we learn from the dynamics of top-up transfers about migration and mobility, and specifically, about irregular migration? While seeking answers to these questions, we introduce several case studies, concerning the sending behavior in times of crisis, as well as hard-to-reach migrant groups, two domains in which existing data collection and analysis practices have severe limitations.

Our contributions are multiple. We first provide a systematic analysis of the determinants and influencing factors for top-up transfers. Then we provide a number of related case studies to illustrate the potential of top-up transfers. We investigate the relationship between top-up transfers and migration statistics by correlating migrant stocks with inflow and outflow of airtime top-ups for each country. We show that the migrant stocks are in significant and high correlation with top-up inflows in most countries. Then, we compare top-up transfers to official remittance flow statistics as collected by the central banks of different countries and the World Bank.

Finally, we investigate the impact of the Covid-19 pandemic on top-up flows. The influence and impact of Covid-19 has been studied from multiple aspects [12]. In particular, the location and mobility of people was important to understand the dynamics of the epidemic [19]. Big data sources can also provide indicators that link human mobility and socio-economic activity [40, 43, 47], both of which are important for studying the effects of the pandemic. Our study contributes insights into mobile remittance behavior during the pandemic, as well as providing a way of identifying broader mobility patterns of migrant communities under certain assumptions.

Our findings suggest that when combined with external data sources, top-up transfer data can help us to grasp the short-term impact of crises or important events on remittance behavior. Additionally, top-up data can complement official data sources by providing insights into certain hard-to-reach migrant groups, and on remittance corridors, which cannot be monitored effectively by international organizations. Our study is the first contribution that provides an in-depth analysis of factors that drive international airtime top-up transfers, as well as a number of potential use cases where this new data source may be employed to complement other data sources.

The rest of the paper is structured as follows. In Sect. 2, we discuss the related work. Section 3 describes the data sources used for the analyses in the paper. Section 4 is a detailed analysis of the factors influencing top-up transfers, and includes an assessment on the effect of Covid-19 on such transfers. Section 5 provides two case studies about what top-up data can do for migration studies, dealing with a spatiotemporally granular visualization of migrant distributions in a country, and revealing migrant presence for corridors missing from official statistics, respectively. Section 6 provides a short discussion of our findings, as well as the ethical and privacy aspects of this work. Section 7 concludes the paper.

## Related work

“Digital remittances” are person-to-person transactions using digital payment methods either at the sender or receiver end of the transaction [6], while “mobile remittances” broadly refer to migrants’ financial transfers sent or received through mobile phones [46]. Airtime top-up transfers can thus be seen as digital, mobile remittances, and form a small part of the total remittances to a country [4, 6, 41, 50].

While there is a broad literature on remittances, little is published in the literature about airtime top-up transfers, due to the difficulty of procuring data on the subject. Individual airtime purchases can be obtained from a mobile telecommunications company, and were used for developing fine-grained indicators of wealth [22], socioeconomic segregation [15], food consumption [18] and employment [48]. In some countries, such as North Korea or Nigeria, airtime top-ups are used for settling small sums and treated as a proxy for cash within the county [26, 49]. In this paper, we are rather interested in the dynamics of international transfers. In this context, top-up data from a single mobile phone operator would only provide information on corridors involving the specific country of the phone operator.

An example analysis of mobile airtime transfers was provided by Blumenstock et al., who explored the impact of an earthquake shock in Rwanda [10]. They found out that such transfers played an important role in coping with the earthquake shock by facilitating communication, and the number of transfers decreased as a function of the distance to the epicenter of the earthquake. They also found that the size and connection density of an individual’s social network increased the likelihood of receiving more transfers.

A related branch of the literature focuses on mobile remittances, which mainly include person-to-to-person mobile money transfers. Such studies look at mobile money purchase and usage patterns through national household surveys [36, 42], and via data obtained by partnership with mobile money companies [8, 24].^1^ A series of studies analyzed the impact of mobile money usage on remittance sending behavior with micro-level data, and surveys done with users of M-PESA, a mobile money service offered by Safaricom [24, 32, 35]. In one of these early studies, Morawczynski and Pickens observed that the users of M-PESA tend to send remittances in smaller amounts and more frequently than cash remittance senders [35]. Airtime top-ups that we analyze in this paper show similar patterns.

The findings of empirical studies strongly suggest that mobile money adaption increases the likelihood of sending and receiving more remittances, in some cases increasing internal migration [8], and providing resilience to users in face of shocks [8, 10]. As airtime top-up transfers are meant to be used as mobile phone credits, they might facilitate information flows between migrants and their networks in home countries. This effect was demonstrated by Batista and Narciso [8] in a randomized field experiment, in which the treatment group was provided with free top-up calling minutes every month for six months. The findings of the experiment suggested that the treatment group sent remittances in a larger amount, and they sent it to a greater number of individuals residing in their home countries [8].

As can be seen from these studies, top-up transfers, just like other forms of remittances, can provide a glimpse into social ties between people at different locations, as well as how these ties are activated over time and in response to events that may drive migration. These insights can change depending on the granularity and availability of the data sources.

## Data sources

The World Bank estimates bilateral remittances for 214 countries and territories. According our analysis, 12,642 corridors are estimated to have nonzero remittance flows between them. We use a dataset of top-up transactions generated and stored by a leading top-up company. The full database contains data for more than 4000 corridors, collected between 01/01/2020 and 12/31/2020. We filtered out the corridors that have less than 5000 airtime transactions per year and ended up with 630 country corridors for our analysis. For the year 2020, 220 M transactions are recorded.^2^ The company is referred to as one of the main actors in the international airtime top-up industry. Due to the fast growing market, it is not possible to know the exact market share of the company. As we also discuss in Sect. 6, we do not access the raw data during the analyses. Instead, the data are spatiotemporally aggregated within the servers of the top-up company, and only the total daily volumes for each corridor are processed. The total monetary value of transfers is not included in aggregation; hence, we only observe the number of total transfers. This way, it is impossible to reach individual-level conclusions (such as wealth indicators per customer), or to jeopardize the privacy of individuals.

**Fig. 1:**
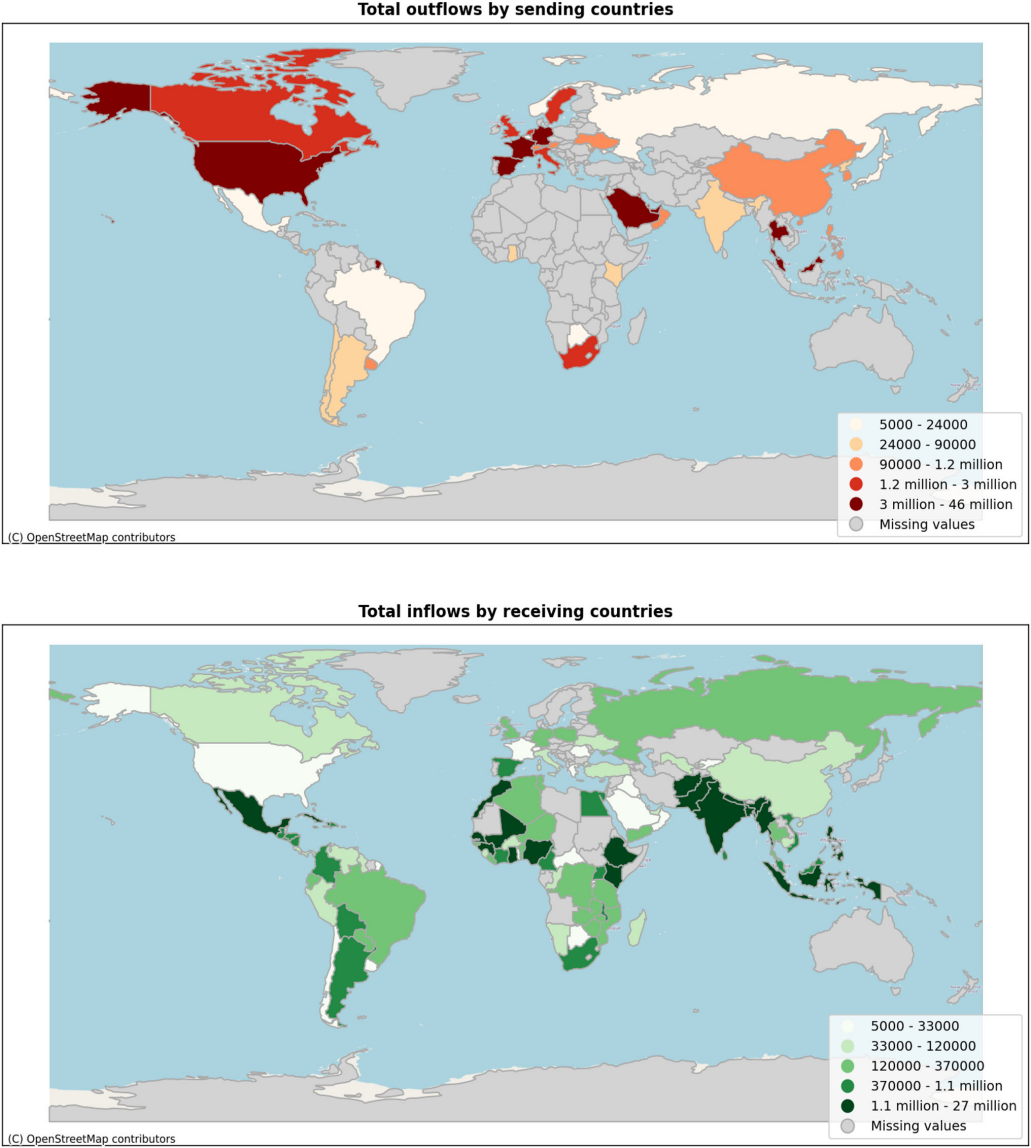
The top figure shows the distribution of the sum of top-up outflows for 2020 per sending country. The bottom figure shows the same data from a receiving country perspective. The five quantiles of the distributions are shown by color intensity, figure best seen in color

The raw data consist of a long list of individual transactions, each registered with a timestamp, unique identifier, amount of transaction, the currency of transaction, the countries of the sender and receiver, and the partner companies who are involved in the transaction. The dataset was first anonymized (i.e., customer and partner information removed) and then aggregated daily and at the corridor level, internally. In total, there are 46 sending and 104 receiving countries. Figure 1 shows the sending and receiving countries by top-up activity.

International airtime top-up data are a unique mobile phone data source. Usually data are collected domestically by one mobile network operator (MNO). International airtime transactions data are collected by B2B companies, which curates data from its partner MNOs. It is one of the rare sources that are useful for developing indicators on international migrant stocks and international remittances.

To better understand the airtime top-up transfer data, we use various external datasets. These are:

1. GDP-related indicators, bilateral import/export flows, inflation numbers per country from International Money Fund (IMF)
2. World development indicators from the World Bank,^3^
3. Bilateral migration stocks from the World Bank^4^
4. Bilateral refugee stocks from the United Nations High Commissioner for Refugees (UNHCR)^5^
5. Economical indicators from the Research and Expertise on the World Economy (CEPII - Centre d’Études Prospectives et d’Informations Internationales)^6^
6. Facebook Social Connectedness Index (SCI) [7]
7. Google community mobility reports [21]
8. Italian National Institute of Statistics (ISTAT)^7^
9. Oxford University indices on country policies around COVID-19 [51]

In Table 1, we share the descriptive statistics on the variables used in this study and provide a source for each.

We would like to give a short description of the indices that are used in this study. Social Connectedness Index calculated by Facebook measures the intensity of connectedness between two countries, as the ratio between the number of friendship connections between country *i* and *j*, and the multiplication of the number of accounts in country *i* and in country *j*. This index has been used in migration studies to calculate migrant stocks [20] and flows [34], and just like remittances, provides insights into the diaspora. Mobile connectivity index is calculated by GSMA, and it measures the performance of countries in terms of mobile internet adoption. Google community mobility reports calculate movement trends over time for different categories such as pharmacies and groceries, parks, retail and recreation, workplaces, and residential. Lastly, stringency index, government response index, containment and health index and economic support index are all developed by Oxford University for assessing effects of Covid-19-related policies.

**Table 1:** The list of all variables used in estimations and analyses. Time is indexed by *t*

Variable	Description	Source
Daily transaction $\hbox {count}_{{ijt}}$	The count of daily transactions from sending country (*i*) to receiving country (*j*)	DT One
Scaled $\hbox {SCI}_{{ij}}$	The social connectedness index of each country-pair	Facebook
Mobile connectivity $\hbox {index}_{{i}}$	The mobile internet connectivity index for each receiving country *j*	GSMA
$\hbox {Residential}_{{it}}$	Mobility trends for residential areas in country *i*	Google
$\hbox {Workplace}_{{it}}$	Mobility trends for workplace areas in county *i*	Google
$\hbox {Retail}_{{it}}$	Mobility trends for areas like restaurants, cinemas, museums etc. in country *i*	Google
$\hbox {GCI}_{{it}}$	Government response index in country *i*	Oxford
$\hbox {CHI}_{{it}}$	Containment and health index in country *i*	Oxford
$\hbox {SI}_{{it}}$	Stringency index in country *i*	Oxford
$\hbox {ESI}_{{it}}$	Economic support index in country *i*	Oxford
Common $\hbox {Border}_{{ij}}$	Sharing common border	CEPII
Common $\hbox {Language}_{{ij}}$	The official language of both countries	CEPII
$\hbox {Colony}_{{ij}}$	The country-pair has a colony past	CEPII
Common $\hbox {colony}_{{ij}}$	Two countries that have been colonized by the same power	CEPII
$\hbox {Distance}_{{ij}}$	The distance between the capitals of each country-pair	CEPII
$\hbox {GDP}_i$	The GDP value for sending countries	IMF
$\hbox {GDP}_j$	The GDP value for receiving countries	IMF
Growth $\hbox {GDP}_i$	The growth GDP values for sending country	World Bank
Growth $\hbox {GDP}_j$	The growth GDP values for receiving country	World Bank
$\hbox {Inflation}_{{i}}$	The inflation rate in sending country	IMF
$\hbox {Inflation}_{{j}}$	The inflation rate in receiving country	IMF
Credits of private $\hbox {sector}_i$	The credits of the private sector in sending country (proportional to GDP)	World Bank
Credits of private $\hbox {sector}_j$	The credits of the private sector in receiving country (proportional to GDP)	World Bank
Dependency $\hbox {ratio}_j$	Age dependency ratio in receiving country (% of working-age population)	World Bank
$\hbox {Exports}_{{ij}}$	Exports from sending country (*i*) to receiving country (*j*)	IMF
$\hbox {Imports}_{{ij}}$	Imports of country i from country *j*	IMF
Youth $\hbox {Unemployment}_{{i}}$	Unemployment of people between age 15 and 24 in country *i*	World Bank
Migrant Stocks $\hbox {estimates}_{{ij}}$	Migrant stocks from country *j* in country *i*	World Bank
Bilateral Remittance $\hbox {Flows}_{{ij}}$	Official statistics of remittance flows	IMF
Refugee $\hbox {Stocks}_{{ij}}$	Official statistics on total number of refugees	UNHCR

## Determinants of top-up transfers

While it is well known that migrants form a major user group for top-up transfers, we investigate this relationship empirically in Sect. 4.1 and then focus on the determinants of top-up transfers in Sect. 4.2 with a gravity model. In Sect. 4.3, we evaluate the specific effects of Covid-19 on the top-up behavior.

### Top-up transfers and migration

In theory, anyone can send airtime top-up transfers between two countries, but the main user base of the top-up company is known to consist of migrant workers. To understand the relationship between airtime top-up transfers and migrants, we investigate a series of correlations between migrant stocks and top-up transfers.

For bilateral migration stocks, we use data from the World Bank, which shares bilateral stocks of 214 countries and territories. The bilateral top-up transfer data we analyze in this paper include 630 pairs of countries, since we did not include country corridors with less than 5000 airtime transaction counts per year.

Many country corridors with a history of migration records do not engage in airtime top-up transfers in our dataset, but this may have many reasons. The more interesting cases are country corridors without any migration record in the World Bank databases that are engaging in airtime top-up transfers.

Since the latest data release on bilateral migration stock estimates by World Bank was in 2017, we first check whether it is representative for 2020 as well. We look at the growth rate in the bilateral migrant stocks between 1990 and 2015 [2], and the bilateral refugee stock differences between 2017 and 2020, as declared by UNHCR [53]. Unless there is a refugee crisis, migration stock rates grew steadily in the data shared by Abel and Cohen [2]. Except for a few corridors, the bilateral refugee stocks did not change significantly for the country corridors in the top-up dataset.^8^ Therefore, we assume that the World Bank data on bilateral migration stocks collected in 2017 reflect 2020’s bilateral migration stock with a small margin of error for the country corridors in our data set.

**Fig. 2:**
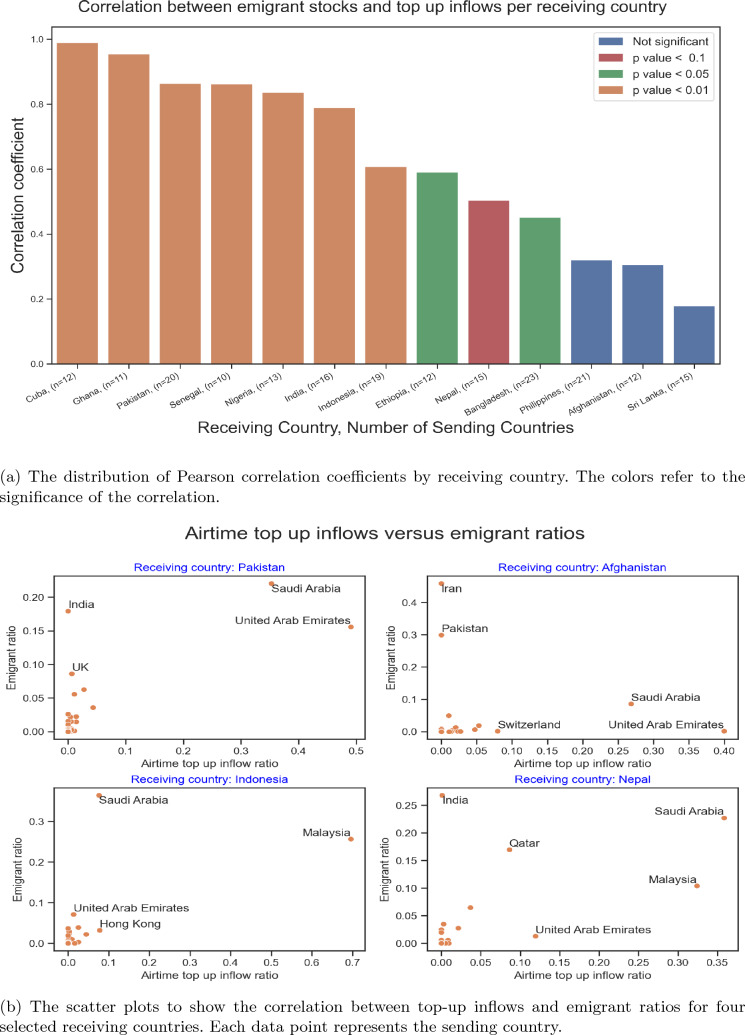
Correlations between emigrant stock and top-up inflow ratios per receiving country

Our correlation analysis looks at the relationships between the top-ups flows and the corresponding migrant stocks. In Fig. 2a, we visualize the distribution of correlation coefficients between emigrant stocks and top-up inflows for each receiving country. The color is based on the level of significance. A country may have multiple groups of migrants, and it can be both sending and receiving remittances. The majority of receiving countries have less than five top-up senders, and only 13 countries have more than 10 senders. Although there is no agreement, less than six data points are considered very low for meaningful correlation analysis [3], so we decided to document correlations with at least ten data points.

We see a high (and significant) correlation between emigrant stocks and top-up inflows. Two-thirds of all receiving countries in Fig. 2a are significantly correlated with migrant stock with a Pearson correlation coefficient higher than 0.5 and *p* value less than 0.01. These ratios hold with correlations less than ten observations as well. On the top of the list, we have countries such as Cuba, Ghana, and Pakistan. Figure 2b gives a more detailed picture for four receiving countries, namely Pakistan, Indonesia, Afghanistan, and Nepal, respectively.

**Fig. 3:**
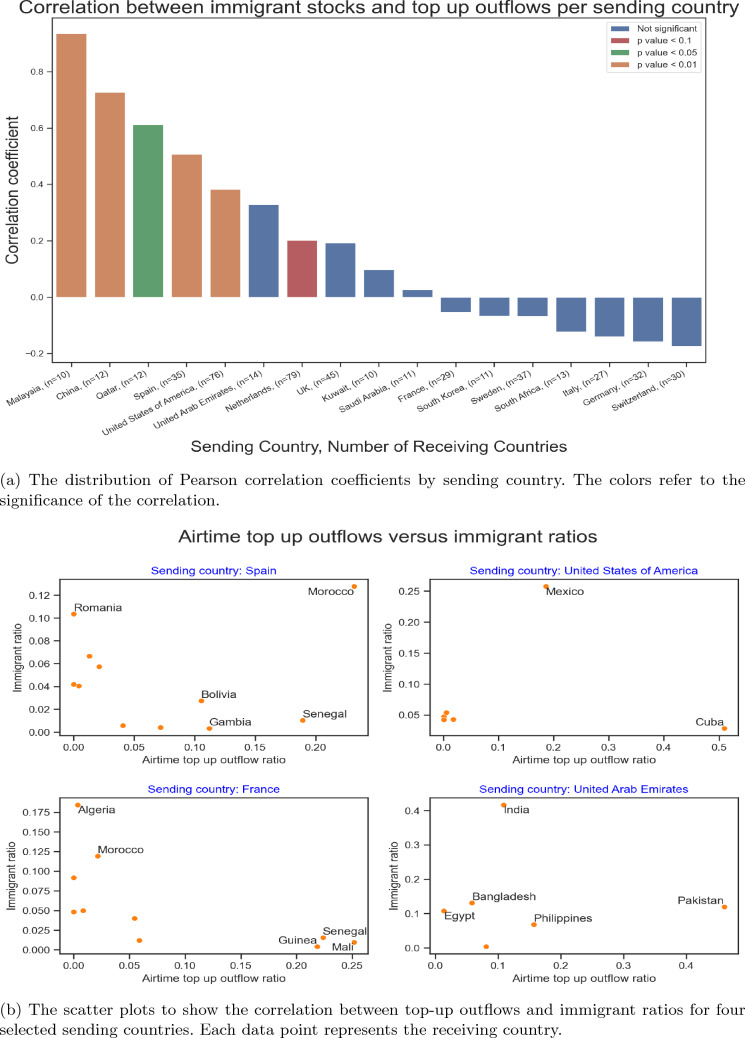
Correlations between emigrant stock and top-up inflow ratios per sending country

A similar result is obtained for the distribution of the correlations between immigrant stocks and top-up outflows per sending country (Fig. 3). For countries with a small number of observations, the correlation coefficients in Fig. 3 may be sensitive to outliers, and the results should be carefully interpreted. The points that appear as outliers are typically important corridors. Also, there are cases where the removal of outliers increases the correlation coefficient, but these may well be due to the small numbers of observations. There are also some biases in the top-up data that result in low correlations. As an example, in Western Europe, sub-Saharan African immigrants use services much more than Northern African immigrants, although the immigrant stocks are much higher for Northern Africans in many sending countries (e.g., Spain and France). In Canada and Sweden, Cuban immigrants dominate the outflows, whereas there are other more populous immigrant groups that are not as active in sending top-ups. Similarly, most Gulf countries have Indians as the largest immigrant group, but they send fewer top-ups than immigrants from Philippines and Ethiopia. Singapore, on the other hand, has a high number of top-up flows to Mexico without any significant migration, because some clients are domiciled in Singapore while operating in Mexico. Such distortions of the correlations between immigrants and top-up outflows are common, and country-specific expertise and qualitative insights are necessary to provide a more healthy picture. Later in Sect. 5.1 we illustrate this in the case of Italy.

These observations confirm empirically that migrants play an important role in generating top-up transfer flows.

### Gravity model for determinants of top-up transfers

The literature on the determinants of remittance focuses distinctively on macro-level and micro-level factors [13, 39]. Micro-level studies [28] developed hypotheses ranging from pure self-interest to pure altruism to explain the motivations of remittance behavior. On the other hand, macro-level studies show that fluctuations in exchange rates between sending and receiving country currencies, interest rate, inflation differentials, and natural disasters impact the remittance flows [39, 56]. The macro-level and micro-level determinants of international airtime top-ups have not yet been investigated, but as discussed before, Blumenstock et al. analyzed the micro-level determinants of domestic airtime remittances in their study on Rwanda [10].

We use a gravity model to explore the determinants of international airtime remittances. Gravity models are popular empirical methods, especially because of their success in estimating international trade flows [17], and they are also frequently used for estimating migration [14, 16, 25, 27], and remittance flows [1, 29, 30]. We focus on the macro-level determinants of international top-up flows.

The annual international airtime top-up transfers are modeled with the following gravity equation:

1 $$\begin{aligned} \log (T)_{ij}= & {} \beta _{0} + \beta _{1} \log (\hbox {GDP})_{i} \\ \nonumber{} & {} +\beta _{2} \log (\hbox {GDP})_{j} + \beta _{3} \log (D)_{ij} \\ \nonumber{} & {} +\beta _{6} X_{ij} + \beta _{6} Y_{i} + \beta _{7} Z_{j} + \alpha _{i} + \gamma _{j} + \epsilon _{ij}, \end{aligned}$$

where $\log (T)_{ij}$ refers to the total annual airtime top-up transactions between sending country *i* and receiving country *j*, expressed in log form. For the main specification, we used the gross domestic product (GDP) of both sending and receiving countries, ${D}_{{ij}}$, the distance between the sending and receiving countries, and ${M}_{{ij}}$, the migrant stock in the sending country from the receiving country. ${X}_{{ij}}$, ${Y}_{{i}}$, and ${Z}_{{j}}$, refer to a set of control variables, at the corridor, sending country, and the receiving country levels, respectively. $\alpha _{i}$ and $\gamma _{j}$ refer to the fixed effects, and $\epsilon _{ij}$ refers to the error term. The full set of variables are listed in Table 2.

**Table 2:** Gravity model estimations

Independent variable	Dependent variable: daily top-up transfers from sending (*i*) to receiving country (*j*)						
	(1)	(2)	(3)	(4)	(5)	(6)	(7)
Log Distance$_{ij}$	0.122***	0.236***	0.224***	0.239***	0.295***	0.344***	0.064
	(0.047)	(0.047)	(0.050)	(0.053)	(0.053)	(0.087)	(0.102)
Log migrant stocks from *j* in *i*	0.187***	0.089***	0.174***	0.166***	0.126***	0.128***	0.155***
	(0.017)	(0.020)	(0.016)	(0.017)	(0.018)	(0.031)	(0.028)
Log GDP$_{i}$	$-$ 0.121**	0.141***				0.012	
	(0.043)	(0.050)				(0.092)	
Log GDP$_{j}$	0.098**	0.338***				0.139	
	(0.041)	(0.045)				(0.084)	
Log GDP per capita$_{i}$	0.056	0.042	$-$ 0.031	$-$ 0.001	$-$ 0.056	0.067	
	(0.086)	(0.090)	(0.090)	(0.091)	(0.090)	(0.162)	
Log GDP per capita$_{j}$	$-$ 0.359***	$-$ 0.578***	$-$ 0.259***	$-$ 0.594***	$-$ 0.447***	$-$ 0.265	
	(0.066)	(0.067)	(0.056)	(0.146)	(0.066)	(0.188)	
Log Scaled SCI$_{ij}$		0.376***	0.250***	0.278***	0.320***	0.333***	0.451***
		(0.045)	(0.043)	(0.044)	(0.044)	(0.075)	(0.077)
Mobility Index country$_{j}$				0.026**		$-$ 0.020	
				(0.010)		(0.016)	
Common border$_{ij}$			$-$ 0.527	$-$ 0.547*	$-$ 0.747**	$-$ 0.189	$-$ 0.583
			(0.330)	(0.329)	(0.327)	(0.385)	(0.365)
Common Official Language$_{ij}$			$-$ 0.169	$-$ 0.143	$-$ 0.173	0.028	$-$ 0.089
			(0.157)	(0.156)	(0.153)	(0.210)	(0.178)
Colony$_{ij}$			$-$ 0.371	$-$ 0.359	$-$ 0.324	$-$ 0.340	$-$ 0.289
			(0.226)	(0.226)	(0.225)	(0.274)	(0.246)
			0.228	0.170	0.074	$-$ 0.557*	$-$ 0.506**
Common colony$_{ij}$			(0.203)	(0.203)	(0.201)	(0.290)	(0.255)
Inflation$_{i}$						$-$ 0.002*	
						(0.001)	
Inflation$_{j}$						0.006	
						(0.066)	
Youth unemployment$_{i}$						$-$ 0.004	
						(0.008)	
Youth unemployment$_{j}$						$-$ 0.006	
						(0.007)	
GDP growth country$_{i}$						$-$ 0.010	
						(0.029)	
GDP growth country$_{j}$						$-$ 0.008	
						(0.016)	
Private sector credits country$_{i}$						0.001	
						(0.002)	
Private sector credits country$_{j}$						$-$ 0.006**	
						(0.002)	
Dependency ratio country$_{i}$						$-$ 0.026***	
						(0.010)	
Dependency ratio country$_{j}$						$-$ 0.008	
						(0.006)	
Log exports$_{ij}$					0.154***	0.144	$-$ 0.011
					(0.031)	(0.063)	(0.067)
Constant	11.060***	$-$ 3.745*	7.473***	8.066***	5.463***	1.806	5.168***
	(1.515)	(2.180)	(1.254)	(1.305)	(1.302)	(3.737)	(1.706)
Fixed effects	No	No	No	No	No	No	Yes
Observations	625	569	569	567	562	414	562
Adjusted *R*-squared	0.217	0.332	0.278	0.285	0.311	0.326	0.531
*F*-statistic	29.81	41.35	25.25	23.60	26.37	9.69	5.632

For each of the variables, the coefficient is given with the std. error in parenthesis. *$p<0.1$; **$p<0.05$; ***$p<0.01$

All variables are collected and summarized for 2020, except bilateral migrant stocks, for which the latest available data is compiled in 2017. For bilateral migrant stocks, although there are only minor changes in a few country corridors, we updated the migrant numbers with the difference in the refugee stocks between 2017 and 2019.

In most of the specifications, we included other country level variables for sending and receiving countries, and corridor level variables to explain the top-up transactions in 2020. We also included country-specific fixed effects in one of the regressions, which is a commonly used panel data method in social sciences. We estimate Eq. 1 in column (1) of Table 2. We extended these baseline estimates including more variables and fixed effects in six additional models, shown in columns (2)–(7).

The number of observations varies based on the availability of the independent variables in each column. Originally, there were 630 top up corridors included in the analysis. GDP-related variables are missing for Venezuela and North Korea. Similarly, the SCI index was not calculated for 61 country corridors in our data set. There are also some missing variables for the inflation and private sector credit variables.

Table 2 clearly illustrates the significance of migrant stocks, social connectivity (as indicated by SCI), as well as the GDP of the receiving country as significant factors. In the first six columns, we also found a positive association between distance between countries and airtime top-up transfers. But after we include country-level fixed effects, this positive association disappears, whereas the common border variable becomes significant. Therefore, the positive influence of distance might be due to the low amount of top-up flows between neighboring countries. Furthermore, some actively sending or receiving countries with distant top-up partners might be causing a bias in the distance variable. However, even if there are more transactions between countries that are more distant, this may not be the case for a specific origin or destination.

Migrant stock and the social connectedness index for a sending & receiving country pair both have a significant and sizable effect on airtime top-up flows in all specifications. As we are doing cross-sectional analysis, we cannot look at the impact of many country level variables with fixed effects. The GDP per capita of the receiving country has a negative relationship with the airtime top-up transfers for most of the specifications. There is no strong association between having a common official language, having a colonial past, or being part of the same colony and top-up airtime transactions. In some cases, adjacency between the countries and common colony past have significant negative impact. The result of some features like adjacency yields negative coefficients in some studies [29], like ours, and positive coefficients in others [33], depending on the sample and the data. In our case, the findings make sense, as most of the international flows still occur between non-neighboring rich and poor countries.

It is important to consider outliers in such an analysis. Some corridors like USA–Cuba, USA–Mexico, or Indonesia–Malaysia can be considered as outliers because the total airtime flow between these corridors is a few times higher than the rest of the corridors. Moreover, Singapore has a high volume of outflows to some countries (like Mexico and Cuba) due to business-related transactions, and not due to transfers made by users. However, the results were not sensitive to these outliers, so we did not exclude them from our analysis here.

The results of the gravity model analysis, combined with the correlation analysis, validate that migrants and their networks play a crucial role in generating international top-up flows. Receiving country demand seems to be the main driver of the flows. If the demand is high in the receiving country, the emigrants from that receiving country send more top-ups around the world. Sending country is also influential on top-up flows in terms of volume because of the type of immigration. There are more flows in countries, in which there are many temporary migrants or migrant workers. Notoriously, Gulf countries, which host millions of migrant workers, are the most active top-up sending countries. Nevertheless, we must note that although we observe high and significant correlations in terms of the relationship between migrants and top-up flows, explanations for a particular country corridor may have idiosyncratic issues.

### Effect of Covid-19 on top-up transfers

There are several additional factors influencing top-up transfers from migrants. For example, national and religious holidays typically increase the amount of remittances for a brief duration. The Covid-19 pandemic has been an influential factor for large scale human behaviors. The pandemic, as well as other disasters, creates conditions of vulnerability, which are expected to increase money and other types of remittances to the vulnerable populations. We focus on Covid-19 in this section.

To track the impact of the Covid-19 pandemic in each corridor, we use four indices developed by Oxford COVID-19 Government Response Tracker [51]; Government Response Index, Containment and Health Index, Stringency Index, and Economic Support Index.^9^ The dataset contains information for more than 180 countries. In addition, we used Google Mobility Trends [21], which shows mobility trends in 135 countries for various place categories like residential areas, workplaces, retail, and recreation areas, and obtained similar results.

In our analysis, we use time-specific and country-specific effects to observe the impact of indices on corridor-level airtime top-up transactions. We hypothesize that top-ups were impacted positively by the lockdown conditions in receiving countries and negatively by the lockdown conditions in sending countries. We test our hypothesis with the following model:

2 $$\begin{aligned} \log (A)_{ijt} = \beta _{0} + \beta _{1} I_{it} + \beta _{2} I_{jt} + \gamma _{i} + \gamma _{j} + \pi _{t} + \epsilon _{ijt}, \end{aligned}$$

where $A_{ijt}$ refers to the daily airtime top-up transaction between receiving country *j* and sending country *i* on day *t*. Independent variables $I_{it}$ and $I_{jt}$ refer to a set of indices and mobility trends in sending and receiving countries, respectively. $\gamma _{i}$ and $\gamma _{j}$ refer to sending and receiving country fixed effects, whereas $\pi _t$ stands for the daily fixed effect. Finally, $\epsilon _{ijt}$ is the error term. These indices and trends vary daily at the country level between February and December, 2020. January was used as the baseline month for Google mobility trends; therefore, it is not included in the analysis.

**Table 3:** Oxford indices based analysis of top-ups flows and the Covid-19 pandemic

Dependent variable: daily airtime top-up transfers from sending country (*i*) to receiving country (*j*)						
	Pandemic phase					
	(I)		(II)		(III)	
Country of Index	Sending	Receiving	Sending	Receiving	Sending	Receiving
Stringency Index	$-$ 0.001***	0.005***	$-$ 0.002**	0.001*	$-$ 0.000	0.000
	(0.000)	(0.000)	(0.001)	(0.001)	(0.001)	(0.001)
Economic Support Index	0.000*	0.005***	0.000	0.001**	$-$ 0.003***	0.001
	(0.000)	(0.000)	(0.001)	(0.001)	(0.001)	(0.001)
Health Containment Index	$-$ 0.001***	0.005***	$-$ 0.001	0.001**	$-$ 0.001	0.000
	(0.000)	(0.000)	(0.001)	(0.001)	(0.001)	(0.001)
Government Response Index	$-$ 0.001**	0.005***	$-$ 0.001	0.001*	$-$ 0.001	0.000
	(0.000)	(0.000)	(0.001)	(0.001)	(0.002)	(0.001)
Day dummies	Yes		Yes		Yes	
Receiving fixed effect	Yes		Yes		Yes	
Sending fixed effect	Yes		Yes		Yes	
No. Obs	71.918		82.112		45.225	
Adj. *R*-squared	0.420		0.443		0.441	

For each of the variables, the coefficient is given with the std. error in parenthesis. *$p<0.1$; **$p<0.05$; ***$p<0.01$

**Table 4:** Google Trends based analysis of top-ups flows and the Covid-19 pandemic

Dependent variable: daily airtime top-up transfers from sending country (*i*) to receiving country (*j*)						
	Pandemic phase					
	(I)		(II)		(III)	
Country of Index	Sending	Receiving	Sending	Receiving	Sending	Receiving
Google Residence Mobility Index	$-$ 0.007***	0.010***	0.006***	0.005***	0.001	0.003
	(0.001)	(0.001)	(0.002)	(0.002)	(0.003)	(0.002)
Google Workplace Mobility Index	0.002***	$-$ 0.003***	$-$ 0.001***	$-$ 0.001***	$-$ 0.000	$-$ 0.001
	(0.000)	(0.000)	(0.001)	(0.001)	(0.001)	(0.001)
Google Retail Mobility Index	0.003***	$-$ 0.003***	$-$ 0.001	$-$ 0.002***	0.001	$-$ 0.002***
	(0.000)	(0.000)	(0.001)	(0.001)	(0.001)	(0.001)
Day dummies	Yes		Yes		Yes	
Receiving fixed effect	Yes		Yes		Yes	
Sending fixed effect	Yes		Yes		Yes	
No. Obs	60.006		68.198		48.333	
Adj. *R*-squared	0.425		0.455		0.454	

For each of the variables, the coefficient is given with the std. error in parenthesis. *$p<0.1$; **$p<0.05$; ***$p<0.01$

The results are shared in Tables 3 and 4. All regression models are based on Eq. 2 and include receiving and sending country fixed effects, as well as daily dummies. We divided 2020 into three phases based on information from the World Health Organization (WHO).^10^ The spread of disease occurred largely between February and June 2020, Phase I in our analysis. From July to September, we have Phase II, and Phase III is from October to December 2020.

According to the analysis, in the first phase the main driver of airtime top-ups was a response to the worsening pandemic conditions in receiving countries, but sending countries that are less affected by the pandemic could send more top-ups. In the second phase, virus cases were already present in all continents. The direction of the effect flips for mobility trends for sending countries, and most of the policy indicators lose significance, except for the stringency index. During this time, the effects are significant in the same direction, with a smaller size of receiving countries.

In the last phase, there is almost no association between the policy indicators, mobility trends and airtime top-up transfers. These results show us that the top-up sending behavior exhibits an adjustment period with a large response to changes in the policy environment and mobility restrictions in the beginning of the pandemic, whose effect decreased slowly throughout the year. These results also confirm that the receiving country situation was an important driver of airtime top-ups during the pandemic.

## Case studies

Having dealt with the determinants of top-up transfers, we now illustrate the potential contributions of top-up transfer data for migration and mobility analysis on two case studies. Using airtime top-up transactions for predicting migration stocks is challenging as it requires us to know the top-up sending behavior of migrant groups in the sending country. Firstly, some migrant groups are disinterested in airtime top-ups, secondly the frequency of top-up sending behavior changes by migrant group. Instead, we use airtime top-up transactions as an indicator of the presence and distribution of migrants. In Sect. 5.1, we show that if the top-up purchase locations are known (e.g., aggregated at the city level), top-up data can serve as a good proxy for the real-time distribution of migrant stocks within the country. In Sect. 5.2, we demonstrate that top-up data can provide information about the existence of migrant groups that are missing in the official statistics.

### Case study I: distribution of migrant groups

Airtime top-up transactions can be considered as strong signals for the location of migrants. Aggregating the data at the city level (i.e., labeling each transaction with the source city) gives useful insights into stock counts per city or country. Official statistics tend to have lags in terms of quantifying the migration stocks. Especially, hard-to-reach groups can be missing in official statistics due to their irregular status or recency of migration. Airtime top-ups are very popular among many hard-to-reach migrant groups, which can be a way to obtain information on the number of such migrant groups living in the sending countries.

Italy, as a country with high quality statistics on migrants, can provide an example case study for the success of top-up data to show timely insights into migrant groups. For this case, we use an additional, city-level indicator for top-up activity in 12 cities. For the first half of 2020, we have aggregated the number of transaction counts sent to different countries from each city of Italy, divided it by the total airtime top-ups sent from that city, and we correlated it with the ratio of official migrant stocks of associated migrant groups in those cities.^11^ The official data on migrant stocks are collected on foreigners living in Italy with a valid residence permit. As with previous correlations, we only included migrant groups with sizable top-up sending activity for a meaningful analysis. As a result, we ended up analyzing 17 migrant groups in Italy. For Romanian, Moroccan, and Cameroonian migrants in Italy, we achieved between 0.7 and 0.8 Pearson correlation coefficients, significant at 1% level. For Bangladeshi, Malian, Guinean, Gambian, Senegalese, Nigerian, and Ghanaian migrant groups, the Pearson correlation coefficient is negative or close to zero, and not statistically significant. For Spanish, Cuban, Pakistani, and Bolivian migrant groups, the Pearson correlation coefficient is between 0.5 and 0.6 and statistically significant at the 5% level.

**Fig. 4:**
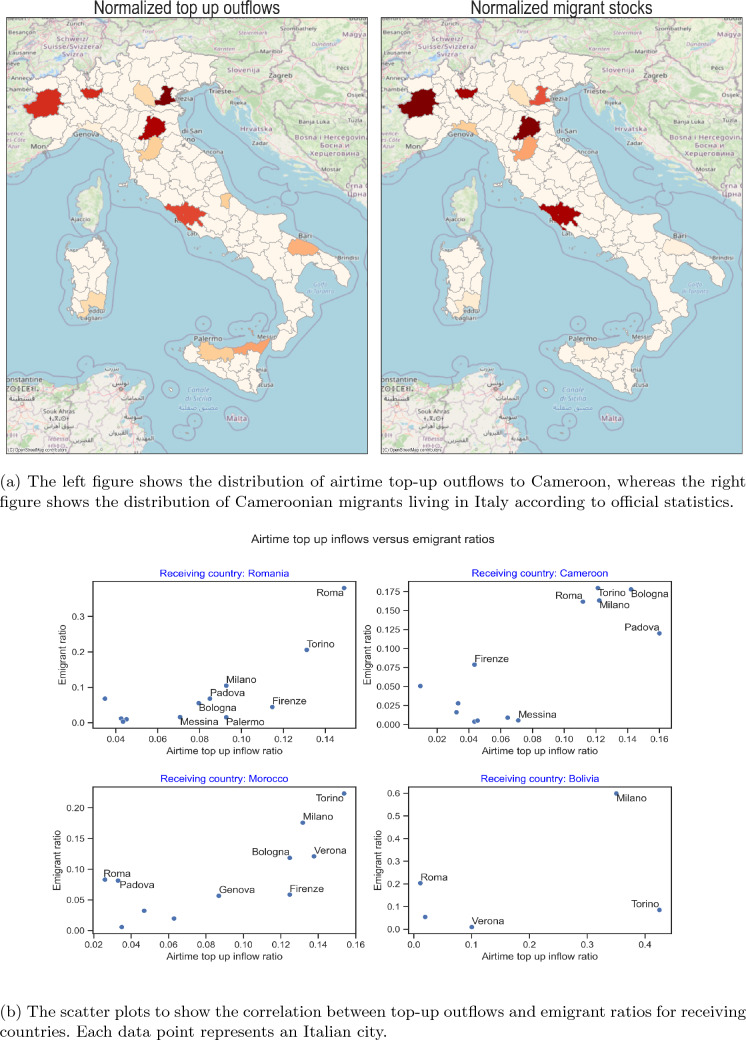
The correlation between emigrant stocks and top-up outflow ratios in 12 Italian cities

In Fig. 4a, we compare the distribution of Cameroonian migrants in Italy based on airtime top-up and official data sources. If we assume that the top-up activity is a good proxy for migrant stocks, we can see that the migrant stocks in Palermo and Messina, major cities in Sicily, and Bari, the capital city of the Apulia region, are underestimated. For almost all migrant groups, the top-up activity suggests a higher level of migrant presence in Sicily and Apulia regions compared to what the official statistics show. For the low correlation coefficient group, the distortion is more enhanced, i.e., the migrant stocks are more seriously underestimated. According to UN statistics on daily refugee arrivals by sea to Italy [54] in 2020, Tunisia and Bangladesh were the top refugee sending countries, with Mali, Gambia, and Guinea following them closely in the list. Sicily and Apulia regions are known for being a common destination for refugee arrivals by sea [54]. The emigrant stocks from Romania are also underestimated in Sicily, although the country is in the European Union, hence Romanians are legally residing in the island. This might be due to the seasonal migration of Romanians to this region [55] for agricultural work in the island.

In Fig. 4b, we show scatter plots for the countries that have the highest Pearson correlation coefficient between emigrants and top-up outflow ratios per Italian city. We see a good level of comparability in big cities in terms of top-up activity and emigrant stocks.

These results suggest that the top-up data could be used to create proxy stock levels in countries where high-quality administrative data on migration are missing. It is not possible to estimate the definite number of migrants missing from the official data with simple comparisons or correlations, because the interest of migrant groups in using top-ups or social media can greatly conflate or deflate the estimates. We note that it is not possible to know whether the top-up activity is associated with irregular or regular migrants with certainty. Also, such data do not show intermediate stops along the migration route. For instance, if we assume that a certain proportion of immigrants from Cameroon came to Italy over France, France could be a target for remittances for Cameroonians in Italy. However, since the average income differences between Italy and France are not as significant as between Italy and Cameroon, and as there are many viable options to mobile money for transferring money between France and Italy, we assume that this practice will be rare. Furthermore, the presence of such transfers will not affect the analysis of the distribution of Cameroon origin migrants in Italy per se, even though it will not be possible to see this intermediate link from top-up data. Consequently, top-up data should be complemented and triangulated with other data sources. When it is used in conjunction with official sources, top-up activity can give rough indication of the presence of refugees and seasonal migrants, which are hard to account for in the official statistics.

### Case study II: missing corridors

**Fig. 5:**
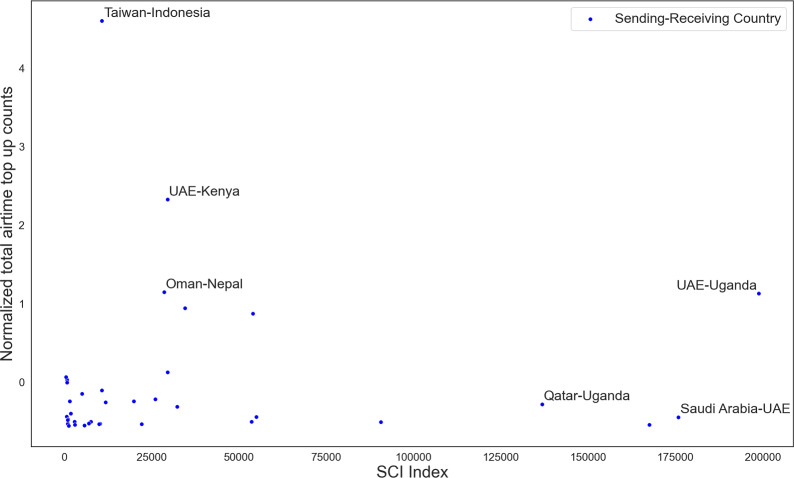
The figure shows the total airtime flows versus SCI index in 2020 for sending–receiving country pairs for which the official statistics show no migrants from the receiving country in the sending country. UAE stands for United Arab Emirates

The official migration statistics are known to have data gaps [9]. This includes corridors for which the migrant count according to World Bank estimations is effectively zero. Airtime top-up transfers for these corridors may tell a different story. To investigate this, we plot the top-up flows (as annual sums) versus the social connectivity index (SCI) in corridors for which World Bank data show no migrants as of 2017 in Fig. 5. In total, there are 36 such corridors.

The reason for these corridors not appearing in the official statistics might be simply due to omissions of some countries. For example, Taiwan is missing as a country from the World Bank data, and it is a known fact that there are many migrant workers from Indonesia in Taiwan [52]. It is also possible that the official statistics do not account for all migrants, depending on the irregularity or recency of migration. For instance, we can see in Fig. 5 a considerable level of top-up traffic between sub-Saharan African countries and Gulf countries. Moreover, the SCI index signals that most of these corridors have a high level of social connectedness on Facebook. This graphic shows activity between country pairs either in airtime top-ups or via SCI index (or both), which reveals missing corridors. More precisely, these findings suggest that for these 36 corridors, we can suspect that the official numbers are underestimating the migrant stocks. As a point of reference, the Kuwait–Sri Lanka corridor has almost the same level of SCI index and top-up flows with the Kenya-United Arab Emirates corridor. The official statistics claim that there are around 40.000 Sri Lankans living in Kuwait.^12^ Similarly, the United Arab Emirates-Uganda corridor can be compared with Oman–Bangladesh in terms of SCI index and top-up flows, and according to the official statistics, there are 276.000 migrants from Bangladesh in Oman. The volume of airtime transactions and SCI index do not give us a number on migrant stocks. Based on average airtime top-up sending frequency of migrant groups, we estimate that there are between 5000 and 10,000 top-up sending Kenyans, and between 3000 and 5000 top-up sending Ugandans in the United Arab Emirates. A higher SCI index does not necessarily indicate more migrants,^13^ but it is an additional evidence on the existence of migrants in the sending country, under the assumption that the country of origin of emigrants corresponds to the receiving country of remittances.

## Discussion

Airtime top-up data are a rich source for complementing the official data sources on remittances and migration, by providing insights into migrants’ remittance behaviors, seasonal mobility, refugee movements, and by complementing official sources on corridors for which available information is limited.

Since the top-up transfer data are privately owned and potentially sensitive, it is difficult to obtain and process. Recent studies have established the legal and ethical good practices in processing sensitive mobile phone based data for migration and mobility studies [44]. In this work, we have used a “privacy by design and default" approach, where raw data stayed entirely at the company servers, and only country- or city-level aggregated figures were shared for analysis. While the original data contain 220 million transactions, we worked with a high level of aggregation for preserving privacy, which resulted in some small sample issues. No personal information (i.e., data that can be used in the identification of individuals) was processed, and the disseminated results are assessed for potential risks to vulnerable groups, such as refugees.

Our investigation demonstrated that the emigrant stocks are strongly associated with the top-up inflows, which suggest that the home-country demand is the driving force behind the airtime top-up flow patterns. In addition to that, for the first time, we showed the determinants of international airtime top-up transfers. The results of gravity model estimations validate our initial observations on the relationship of emigrant stocks to airtime top-up flows. We also found out that larger sending country economies and poorer receiving countries are associated with higher airtime top-up flows, and the Social Connectedness Index (SCI) is a strong determinant of airtime top-up flows.

We showed that daily cross-border airtime top-up transaction counts can be a useful proxy indicator for monitoring hundreds of remittance corridors in real time. One interesting aspect that emerged from the analysis is that the airtime top-up data can be used to measure the short-term impact of country-level or global natural crises to demonstrate the behavior of thousands of migrant groups in the digital space. We found that when the receiving country is facing a crisis, the flow of airtime remittance peaks.

Our findings are in line with the single-country results obtained by Blumenstock et al. [10], who analyzed the impact of an earthquake on airtime top-up flows in Rwanda. We found a significant and positive impact of the Covid-19 pandemic on top-up transfers to receiving countries, which is in line with the small but significant airtime remittance-inducing effect of the earthquake in Rwanda [10]. Secondly, we confirmed the influence of social connectedness between the sender and receiver at the macro-level as well.

As all digital data sources, top-up data we use in this paper have certain biases that need to be taken into account. The data collection is performed in a commercial context, and subsequently, business decisions, operations, and technical issues can cause certain patterns in the data. One of the issues we observed is a sudden start or rupture of top-up flow in some corridors. For instance, in Indonesia, domestic airtime top-up traffic comes to a sudden end in July 2020. Some of these patterns are due to formation of new partnerships or termination of old ones, which can have a considerable impact on a single corridor. Another issue is that some clients might work with users in one country, while being established in another country, as in the case of Singapore and Mexico. Such issues will produce noise in the top-up flow analysis.

## Conclusions

This study is a first foray into the analysis of international airtime top-up flows for migration and mobility. This is a new data source enabled by a business-to-business model, where mobile phone credits are sent internationally as a form of remittances. While this market is currently a small fraction of the global remittance market, it is growing, and it is particularly important for countries without stable and reliable banking solutions.

Our analysis shows that airtime top-up transfers can provide insights into migration corridors at city or country level and permit temporally granular analysis, which is more difficult with official statistics. We have shown that the top-up flows can be used to observe the distribution of migrant groups within a country, or to find out more about corridors with missing official statistics.

Our results also provide a significant first step toward understanding the determinants and influencing factors of international airtime top-up flows. In our analysis, we observe that the demand in the receiving country seems to be correlated with airtime remittance from emigrants, especially in times of crisis, but additional qualitative analysis is required to verify this hypothesis.

Current trends suggest that the importance of such data will increase in the future, as the demand for top-up services are on the rise in many countries. Additional data collection and collaboration with the owners of such data can help to do more granular analysis of airtime remitting behavior at the micro level. Future research should also look at the relationship between remittance flows and airtime top-up flows by using micro-level data and by conducting surveys with users to test for any causal relationships.

## Footnotes

1 A detailed list of micro-empirical studies done with mobile money can be accessed in the work of Aron and Muellbauer [5].

2 To indicate the market size, according to the Global System for Mobile Communications (GSMA) metrics, around 1.180B top-up transactions were registered in 2020. Proportional to GSMA figures, the registered transaction volume we work with might seem low, but GSMA data primarily includes aggregated domestic transfers per region, whereas our data primarily contains international transfers. For GSMA figures, see https://www.gsma.com/mobilemoneymetrics/#global.

3 Data retrieved from: https://databank.worldbank.org/source/world-development-indicators.

4 Data retrieved from: https://databank.worldbank.org/source/globalbilateral-migration.

5 Data retrieved from: https://www.unhcr.org/data.html.

6 Data retrieved from: http://www.cepii.fr.

7 Data retrieved from: https://www.istat.it/.

8 This is simply due to the fact that majority of country corridors, in which large refugee flows took place, do not send or receive airtime top-ups within out dataset.

9 The methodological details and interpretation of these indices are available at: https://github.com/OxCGRT/covid-policy-tracker/tree/master/documentation.

10 The information is available at: https://www.who.int/news/item/29-06-2020-covidtimeline.

11 The data regarding migrant groups per province of Italy are collected by ISTAT in 2020.

12 Kuwait–Sri Lanka and Oman–Bangladesh corridors are not in Fig. 5, as there are migrants recorded by official statistics in these corridors.

13 The SCI index has a tendency to give larger numbers for countries with a small number of Facebook users. The connectivity between small island states tops the list for the SCI index.
